# Successful Removal of a Large Bronchocele

**Published:** 1866-12

**Authors:** Wm. Warren Greene

**Affiliations:** Professor of Surgery in Berkshire Medical College and in the Medical School of Maine


*Successful Removal of a Large Bronchocele.* By WM. WARREN GREENE, M.D., Professor of Surgery in Berkshire Medical College and in the Medical School of Maine.

I have been repeatedly asked by patients, as has every surgeon of much experience, to excise bronchoceles, when small, from apprehension of increased growth, and when large, to relieve deformity or discomfort from weight and pressure, but have never before seen a case in which I should have felt a shadow of justification in using the knife; still I knew such cases did occur, and have often, with myself, discussed the surgical *possibilities* under such circumstances. Upon a careful examination of the books, I inferred that in most instances the knife had been used freely around the tumor, and that in several instances the attempt had been made to remove a *part* only of the morbid growth; and one surgical writer of distinction alludes to a case of this as being remarkable, on account of the uncontrollable hemorrhage, (the patient dying on the table,) when a removal of only a *small portion* of the mass was attempted. Now, each lobe of the thyroid gland is supplied by two arteries usually, sometimes three; no matter how large the goitre, these arteries, however much enlarged, are found entering *the base* of the tumor into which they pass, and their branches ramify through its substance, comparatively few arterial vessels being found upon the surface, which is covered with a network of veins, oftentimes of large size, having extremely thin, delicate walls, and which inosculate freely with those in the substance of the gland. It had occurred to me that in the event of wounding these superficial veins, it might be useless to litigate them, they are so tender, and operations might have failed which would have otherwise succeeded, from the attempt to control the hemorrhage completely during the dissection, in which the knife was perhaps too freely used; and that it was worse than folly to remove a part of a lobe, as almost invariably we should thus open scores of vessels whose bleeding would be uncontrollable. I believed that if they could be removed at all, it must be accomplished by keeping clear of the tumor as far as possible, and rapidly pushing down to the pedicle, which consists mainly of vessels and areolar tissue.

On the 19th of August last, Mrs. Klopf, aged forty-five, a highly respectable and intelligent German lady, residing in Albany, N. Y., consulting me for an enlargement of the right lobe of the thyroid gland; there was a slight enlargement of the left side. She first noticed the tumor twenty-six years ago, but it had never given her any trouble until within a year and a half, during which time it had rapidly increased to its present size. Its dimensions were now such that the common carotid was crowded behind the posterior edge of the sterno-mastoid muscle, where its pulsations were distinctly felt, and the trachea was pressed firmly to the left side. So great was the pressure upon these organs and the œsophagus, that any attempt to swallow or talk gave her terrible spasms of dyspnœa. She was unable to lie down, and required constant watching during her broken sleep, lest she should die of suffocation. See suffered very much from headache and giddiness, and could not stoop without losing her consciousness. All these symptoms had been for two months rapidly increasing in intensity, and for two weeks almost a daily aggravation had been noticeable. She had recently consulted the leading surgeons of Albany and some other cities in New York, all of whom told her that nothing could be done. At my request my colleagues, Professors Ford, Palmer and Storer, and Drs. Smith, Brewster and others, examined her; and all agreed that a few days at most would terminate her existence, while the thread was liable to snap at any hour. The patient was already aware of her imminent danger, and her only question was as to the *possibility* of relief. I told her in all probability the removal of the growth would be found impracticable; and that even if she survived the operation, which was not likely, the chances were a hundred to one that she would die of secondary affections; but still there was a bare possibility of success, and that while I would by no means advise an operation, yet if she, being fully aware of all the facts, insisted upon taking this desperate chance, I would undertake the removal of the tumor. I was happy to have my own views as to the propriety of this advice endorsed by Dr. Storer. She immediately decided upon its being done, and at once. Accordingly, in the presence and with the assistance of Prof. H. R. Storer, Dr. F. K. Paddock, and several students, I made the operation in the following manner: the patient, being etherized, was placed in the ordinary position for ligation of the carotid, and a single straight incision was made over the tumor, extending from the inferior maxilla to the clavicle. The anterior external jugular vein, which ran close by the line of the incision, was not injured. The sterno-mastoid, which was spread over the mass like a thin riband, and the several fasciæ, were successively divided upon a grooved director, and the areolar tissue with the knife and fingers, the handle of the scalpel being employed much more freely than the blade. On raising upon the director the little thin layer of fascia immediately investing the tumor, several veins were wounded and bled profusely; this was controlled by the fingers of an assistant, and the delicate envelope carefully reflected from the gland; but although this was done with the utmost caution and gentleness, several other veins were ruptured. I now found that the entire growth was completely covered with a network of these vessels; and so thin and tender were their walls that the forceps tore, and the ligature cut their coats; and now, although the blade of the knife had not touched the surface of the tumor, so many of these veins were opened, that in spite of all the pressure that could be made, the hemorrhage was fearful. I now rapidly separated the areolar attachments, and in a few seconds was at the pedicle, which I found containing three large arteries whose pulsations were very distinct, and which were my guides for dividing the pedicle into three parts, which I also accomplished with the fingers. I immediately tied each third with a ligature composed of eighteen strands of saddler's silk, *saturated* with wax and *loosely twisted.* As I drew the last chord all hemorrhage instantly ceased. The pedicle was carefully divided close to the goitre, and it removed. During the dissection I found at one point the tumor quite firmly adherent to the sheath of the vessels; and while separating it, a gush of venous blood indicated the rupture of a large vessel. The finger of an assistant controlled it until the ablation of the bronchocele, when examination proved the internal jugular to be wounded. This was tied with a ligature of three strands of silk loosely twisted; no other vessels needed interference. The entire operation occupied twenty-two minutes.

After carefully sponging the wound and allowing the surface to glaze somewhat, the edges were united by interrupted sutures, water-dressing applied, and she placed in bed. The extremities were cool, and pulse feeble but regular, and less than ninety per minute. She reacted nicely, and passed a more comfortable night than she had for weeks. The after-treatment consisted in perfect quiet, water dressings when agreeable, anodynes *pro re nata;* the exhibition of muriated tincture of iron, twenty drops every four hours, and as much rich broth, gruel, and milk as she would take at regular intervals. For several days there was considerable irritation of the œsophagus and trachea, but not enough to interfere seriously with deglutition or respiration. This passed away, and she recovered without a bad symptom. The last ligature came away on the twenty-sixth day; in another week the wound was entirely healed, and she is now in perfect health and restored to a happy family.

The weight of the tumor was one pound nine ounces avoirdupois.—*Med. Record,* Dec. 1.

